# Comparative Multi-Donor Study of IFNγ Secretion and Expression by Human PBMCs Using ELISPOT Side-by-Side with ELISA and Flow Cytometry Assays

**DOI:** 10.3390/cells4010084

**Published:** 2015-02-11

**Authors:** Jodi Hagen, Ryan Zimmerman, Christine Goetz, Jody Bonnevier, Jeffrey P. Houchins, Kevin Reagan, Alexander E. Kalyuzhny

**Affiliations:** R&D Systems, Inc., 614 McKinely Place, Minneapolis, MN 55413, USA; E-Mails: Jodi.Hagen@bio-techne.com (J.H.); Ryan.Zimmerman@bio-techne.com (R.Z.); Christine.Goetz@bio-techne.com (C.G.); Jody.Bonnevier@bio-techne.com (J.B.); JP.Houchins@bio-techne.com (J.P.H.); Kevin.Reagan@bio-techne.com (K.R.)

**Keywords:** human IFNγ, ELISPOT, ELISA, flow cytometry, human peripheral blood mononuclear cells, PBMCs, side-by-side comparison, multi-donor study

## Abstract

ELISPOT, ELISA and flow cytometry techniques are often used to study the function of immune system cells. It is tempting to speculate that these assays can be used interchangeably, providing similar information about the cytokine secreting activity of cells: the higher the number of cytokine-positive cells measured by flow cytometry, the higher the number of cytokine-secreting cells expected to be detected by ELISPOT and the larger the amount of secreted cytokine expected to be measured by ELISA. We have analyzed the expression level and secretion capacity of IFNγ from peripheral blood mononuclear cells isolated from five healthy donors and stimulated by calcium ionomycin mixed with phorbol 12-myristate 13-acetate in a non-specific manner in side-by-side testing using ELISPOT, ELISA and flow cytometry assays. In our study, we observed a general correlation in donors’ ranking between ELISPOT and flow cytometry; ELISA values did not correlate with either ELISPOT or flow cytometry. However, a detailed donor-to-donor comparison between ELISPOT and flow cytometry revealed significant discrepancies: donors who have similar numbers of IFNγ-positive cells measured by flow cytometry show 2–3-fold differences in the number of spot-forming cells (SFCs) measured by ELISPOT; and donors who have the same number of SFCs measured by ELISPOT show 30% differences in the number of IFNγ-positive cells measured by flow cytometry. Significant discrepancies between donors were also found when comparing ELISA and ELISPOT techniques: donors who secreted the same amount of IFNγ measured by ELISA show six-fold differences in the number of SFCs measured by ELISPOT; and donors who have 5–7-times less secreted IFNγ measured by ELISA show a two-fold increase in the number of SFCs measured by ELISPOT compared to donors who show a more profound secretion of IFNγ measured by ELISA. The results of our study suggest that there can be a lack of correlation between IFNγ values measured by ELISPOT, ELISA and flow cytometry. The higher number of cytokine-positive cells determined by flow cytometry is not necessarily indicative of a higher number of cytokine-secreting cells when they are analyzed by either ELISPOT or ELISA. Our ELISPOT *vs.* ELISA comparison demonstrates that the higher number of SFCs observed in ELISPOT does not guarantee that these cells secrete larger amounts of cytokines compared to donors with lower SFC numbers. In addition, our data indicate that ELISPOT, ELISA and flow cytometry should be performed as complementary, rather than stand-alone assays: running these assays in parallel on samples from the same donors may help to better understand the mechanisms underlying the physiology of cytokine-secreting cells.

## 1. Introduction

IFNγ is a cytokine that plays a key role in innate and adaptive immunity [1,2,3,4,5,6], and this is the reason why accurate assessment of IFNγ levels is of critical importance in evaluating the status of the immune system. There are several widely-used techniques for measuring the production of this cytokine, including ELISPOT [7,8,9,10,11], ELISA [12,13,14,15] and flow cytometry [16,17,18], but each technique alone has its limitations, and therefore, they need to be used in combination for a more accurate understanding of the function of the immune system cells. ELISA measures the total amount of secreted cytokine, but does not measure the number of cells that contribute to its production. Flow cytometry can determine the number of cells that produce IFNγ, but not their secretory activity and the amount secreted by them. ELISPOT quantifies the frequency of IFNγ-secreting cells, but not the number of producing cells nor does it measure the amount of IFNγ secreted from cells. Although it is tempting to speculate that there should be a direct correlation between the number of IFNγ-producing and -secreting cells and the amount of secreted IFNγ, there is no experimental data supporting this speculation.

To address this issue, we analyzed PBMCs isolated from five different donors using ELISPOT, ELISA and flow cytometry assays in a side-by-side comparison study. For some donors, we observed positive correlation, whereas for other donors, such a correlation was negative. The results of our comparison study indicate that an accurate assessment of the status of the immune system towards IFNγ production and secretion cannot be entirely based on just one assay, but requires side-by-side testing using ELISPOT, ELISA and flow cytometry.

## 2. Experimental Section

### 2.1. Cells

Blood samples were collected from five donors who were reported negative for STS (Serologic Test for Syphilis), HbsAg (Surface antigen of the Hepatitis B virus), anti-HBc (Surface antigen of the Hepatitis C virus), anti-HIV-1/2, anti-HTLV-I/II (Human T-Lymphotropic Virus), anti-HCV and HIV-1 and supplied in standard citrate-phosphate-dextrose unit bags (Leukopack, Memorial Blood Centers of Minnesota). Peripheral blood mononuclear cells (PBMCs) from each donor were separated using density centrifugation (500× *g* for 30 min) of 25 mL of blood layered on 20 mL of 1.077 g/mL Ficoll-Paque™ PLUS (Amersham Pharmacia Biotech, #17-1440-02) at 25 °C. After centrifugation, the upper plasma layer was discarded, and PBMCs were transferred into two sterile 50-mL tubes. PBMCs were resuspended in 45 mL of sterile phosphate-buffered saline (PBS: 154 mM NaCl (#BP358-1, Fisher Scientific, Minneapolis, MN, USA), 1.54 mM KH_2_PO_4_ (Fisher Scientific, cat #P285-500) and 2.7 mM Na_2_PO_4_ (#S471-3, Fisher Scientific, Minneapolis, MN, USA), pH 7.4) and centrifuged for 5 min at 500× *g*. The supernatant was discarded, and the pellet was resuspended. Remaining red blood cells were removed by adding 10 mL of a lysing solution consisting of 155 mM NH_4_Cl (#A-0171, Sigma Chemical Co., St Louis, MI, USA), 10 mM NaHCO3 (#11810-025, Gibco-BRL, Grand Island, NY, USA) and 0.1 mM EDTA, pH 7.4 (#E-5134, Sigma Chemical Co., St Louis, MI, USA) and incubated for 5 min at room temperature. After lysing, sterile PBS was added (to reach the 50-mL graduation mark on the tube) to resuspend PBMCs. Tubes were then centrifuged for 5 min at 500× *g*. Supernatants were discarded, and 30–40 mL of RPMI complete medium (Life Technologies, #22400-121) supplemented with 10% heat-inactivated fetal calf serum (GE Healthcare Life Sciences, Pittsburgh, PA, USA, #SH30071.03IR), 1.19 g HEPES (Sigma Chemical Co., #H-3375), 2.0 g of sodium bicarbonate (Gibco-BRL, #11810-025), 3.49 μL of beta-mercaptoethanol (Sigma Chemical Co., St. Louis, MO, USA #M-6250) and 50 mg gentamicin reagent solution (Gibco-BRL, #17750-060) were added to the tubes containing PBMCs. Then, cells were spun down by centrifugation using 50-mL sterile culture tubes at 500× *g* and counted using a hemocytometer to make required dilutions as follows: mix 50 µL of cell suspension with 50 µL of trypan blue dye; add 10 µL of cell suspension to each side of the counting surface; and count the number of cells per the hemocytometer instructions under the microscope using a 20× lens and a phase-contrast condenser. The number of dead cells was determined by counting cells stained with trypan blue.

### 2.2. ELISPOT Assay

Commercially available, ready-to use ELISPOT kits to measure the secretion of human IFNγ (Catalog #EL285, R&D Systems, Inc., Minneapolis, MN, USA) were used in this study. Each kit included a dry 96-well PVDF-backed plate pre-coated with corresponding capture antibodies, a concentrated solution of detection antibodies and a concentrated solution of streptavidin-conjugated alkaline phosphatase, BCIP/NBT (5-bromo-4-chloro-3'-indolyphosphate/nitro-blue tetrazolium) substrate and wash and dilution buffers. Assays were performed according to the protocols included with each ELISPOT kit using streptavidin-alkaline phosphatase and BCIP/NBT detection chemistry, as we previously reported [19,20,21]. PBMCs from five donors were plated (100 µL/well) in triplicate 5 × 10^3^ cells/mL and cultured 18 hours in a humidified CO_2_ incubator at 37 °C in the same ELISPOT plate for better assay consistency. Two wells per donor were dedicated to medium only with PBMCs and medium only without PBMCs as controls for the non-specific formation of spots.

To stimulate the secretion of IFN-γ, PBMCs were incubated in ELISPOT plates in the presence of 0.5 μg/mL of calcium ionomycin (CaI, Tocris Bioscience, Abingdon, UK #1707) mixed with 50 ng/mL of phorbol 12-myristate 13-acetate (PMA, Tocris Bioscience, #1201); these concentrations of mitogens were found optimal to induce maximum stimulation without causing cytotoxic effects (data not shown). Plates were washed using a hand-held 12-channel Nunc™ Immuno washer (Thermo Scientific), and spots were quantified using a QuantiHub V4.1 ELISPOT reader (MVS Pacific, LLC). The activity of IFN-γ-secreting cells was measured by the counting spots formed by IFN-γ secreted from individual cells and designated as spot-forming cells (SFCs). The number of SFCs was calculated by dividing the number of spots by the number of plated cells per well and expressed as a percentage (%). Statistical analysis of SFC counts was performed in Microsoft Excel.

### 2.3. Flow Cytometry Assay

PBMCs (5 × 10^6^) were stimulated with 0.5 μg/mL of calcium ionomycin (CaI, Tocris Bioscience, #1707) mixed with 50 ng/mL of phorbol 12-myristate 13-acetate (PMA, Tocris Bioscience, #1201) 18 h at 37 °C in a CO_2_ incubator. To increase the intracellular accumulation of IFNγ, PBMCs were cultured for 3 h (at 37 °C) in the presence of 3 μM monensin (Sigma Chemical Co., #M-5273) prior to intracellular staining. Resting PBMCs from these donors served as a control. Cells were stained for 10 min with human IgG (1 μg/sample) as a blocking reagent followed by incubation with mouse monoclonal anti-human CD3 epsilon-allophycocyanin (APC)-conjugated antibody (R&D Systems, Inc. #FAB100A) for 20 min at room temperature for surface labeling. Cells were then washed twice with 1× HBSS (Hanks Balanced Salt Solution) (Gibco-BRL, #14175-079) and fixed with 4% paraformaldehyde for 15 min at room temperature. After that, PBMCs were permeabilized with 1× Flow Cytometry Permeabilization/Wash buffer (R&D Systems, Inc., Minneapolis, MN, USA, #FC005) and stained with anti-human IFN gamma-phycoerythrin (PE) (R&D Systems, Inc., #IC285P) for 30 min. Cells were washed with 1× flow cytometry permeabilization/wash buffer and analyzed on a Fortessa™ flow cytometer (BD Biosciences). Flow cytometry results were based on gating at the lymphocyte population. Data analysis was carried out using Flowjo software.

### 2.4. ELISA

ELISA is less sensitive than the ELISPOT assay, and for this reason, we had to use a higher number of cells/mL in ELISA compared to ELISPOT. PBMCs (5 × 10^5^ mL^−1^) were seeded into 25-cm^3^ flasks in 5 mL of RPMI complete. PBMCs were stimulated with 0.5 µg/mL CaI and 50 ng/mL PMA added directly to cells in flasks and incubated in a humidified CO_2_ incubator at 37 °C for 18 h. After the incubation, cells were transferred into the sterile tubes and centrifuged for 5 minutes at 500× *g*. Supernatants were collected, and PBMCs were discarded. Supernatants were stored at 2–8 °C until use (but no longer than 2 h) in a commercially available Quantikine ELISA kit for measuring human IFNγ (R&D Systems, Inc., #DIF50). Assays were performed according to the protocols included with the kit. Plates were analyzed by measuring the optical density of each well within 30 min after finishing the assay using a microplate reader (EMax Endpoint ELISA Microplate Reader, Molecular Devices) set to a 450-nm wavelength. The dynamic range of our commercial ELISA kit is 15.6–1000 pg/mL.

## 3. Results and Discussion

### 3.1. ELISPOT

**Figure 1:**
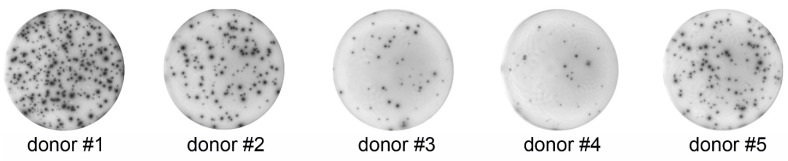
Exemplary images (collected using QuantiHub 4.1 ELISPOT reader) of IFNγ-formed spots by PBMCs isolated from five different donors. The same number of cells from different donors was plated per well and cells were stimulated with the same mitogens for the same period of time. Notice the significant donor-to-donor differences in the spot-forming activity of PBMCs.

The frequency of IFNγ-secreting PBMCs (measured by counting SFCs) stimulated with mitogens ranged from 0.46% to 6.52% and was different for all five donors (Figure 1 and Table 1). No SFCs were detected in the unstimulated control group (data not shown). With regards to IFNγ-secreting activity measured by ELISPOT, donors were ranked as follows: 1 > 2 > 5 > 3 > 4.

**Table 1:** ELISPOT data analysis using a QuantiHub 4.1 ELISPOT reader. In each donor group, spots were counted using identical optical settings and illumination conditions and the same spot-counting software algorithm.

Donor	Spots Per Well ± SD	Spot-Forming Cells (%) ± SD
**1**	326 ± 11.15	6.52 ± 0.23
**2**	155 ± 17.76	3.10 ± 0.36
**3**	47 ± 5.74	0.94 ± 0.12
**4**	23 ± 0.71	0.46 ± 0.02
**5**	143 ± 12.40	2.90 ± 0.23

### 3.2. ELISPOT vs. ELISA

Significant donor-to-donor variations in IFNγ secretion were also observed by ELISA (Table 2, and donors were ranked as follows: 2 > 4 ≥ 5 > 1 > 3. When measured by ELISPOT, Donor #1 showed the strongest IFNγ-secreting activity, whereas this donor was only the fourth highest as measured by ELISA. Donor #4 was the weakest measured by ELISPOT, but was found to be the second strongest when measured by ELISA. Some ELISPOT/ELISA correlation was observed with Donor #2: this was the second strongest measured by ELISPOT and the strongest measured by ELISA.

**Table 2:** ELISA measurements of IFNγ-secreting activity from PBMCs isolated from five different donors. Cells were stimulated with mitogens as described in the Experimental Section. Levels of IFNγ secreted from non-stimulated cells could not be measured, because they were below the detection limits of ELISA (data not shown).

Donor	ELISA (pg/mL) ± SD
**1**	212 ± 4.38
**2**	1449 ± 0.00
**3**	48 ± 2.46
**4**	268 ± 8.19
**5**	266 ± 1.803

Donors #4 and #5 were the same in ELISA (268 ± 8.19 pg/mL *vs.* 266 ± 1.803 pg/mL), but in ELISPOT, Donor #5 displayed six-times more SFCs compared to Donor #4 (2.9% ± 0.23% *vs.* 0.46% ± 0.02%). ELISA showed that the amount of IFNγ secreted from Donor #2 cells was almost seven-times higher compared to Donor #1 (1449 ± 0.0 pg/mL *vs.* 212 ± 4.38 pg/mL), but in ELISPOT, we observed that Donor #2 showed two-times less SFCs compared to Donor #1 (3.1% ± 0.36% *vs.* 6.52% ± 0.23%). Cells from Donor #4 secreted five and a half-times more compared to cells from Donor #3 (268 ± 8.19 pg/mL *vs.* 48 ± 2.46 pg/mL), as measured by ELISA; in ELISPOT, we observed that Donor #4 cells produced two-times less SFCs compared to Donor #3 (0.46% ± 0.02% *vs.* 0.94% ± 0.12%). Cells from Donors #2 and #5 produced almost the same number of SFCs (3.1% ± 0.36% *vs.* 2.9% ± 0.23%), but cells from Donor #2 secreted five-times more IFNγ than Donor #5 cells (1449 ± 0.0 pg/mL *vs.* 266 ± 1.803 pg/mL) (Figure 1, Table 1 and Table 2).

### 3.3. ELISPOT vs. Flow Cytometry

**Figure 2:**
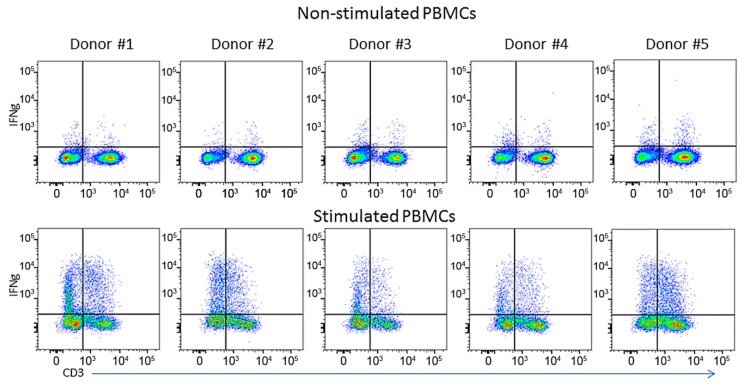
Flow cytometry analysis of IFNγ expression in PBMCs isolated from five donors. Non-stimulated cells were resting PBMCs that served as a control. Stimulated cells were treated with CaI/PMA. Both resting and treated cells were stained for intracellular hIFNγ using the same staining conditions.

As determined by flow cytometry, there were significant donor-to-donor variations in the number of IFNγ-positive cells. In non-stimulated cells, the variability was more pronounced for CD3-negative than for T-cells, but in stimulated cells, a high degree of variability was observed in both CD3-negative and in T-cell populations (Figure 2 and Table 3). The general ranking of donors in the ELISPOT study matched exactly the ranking of donors analyzed by flow cytometry (refer to the “all cells” column in Table 3), and Donor #1 gave the strongest response in ELISPOT and in flow cytometry, followed by Donor #2, which is a strong general correlation of trends of IFNγ secretion and its intracellular expression. Unlike the “all cells” group, the correlation between flow cytometry and ELISPOT data was weaker for stimulated CD3-negative and T-cells (Table 4). When we calculated the fraction of IFNγ-positive cells (measured by flow cytometry), which secreted this cytokine (quantified by ELISPOT), donors ranked as follows: 1 > 2 > 5 > 3 > 4 (Table 5); which precisely matched the donors’ rankings in both flow cytometry and ELISPOT. Although there was a strong general correlation between ELISPOT and flow cytometry, this correlation was not linear. For example, in the “all cells” group, Donors #1 and #2 have almost the same number of IFNγ-positive cells found by flow cytometry, 33.4% and 32.3%, respectively, but the number of SFCs observed by ELISPOT with Donor #1 was more than two-times higher compared to Donor #2: 6.52 ± 0.23 *vs.* 3.1 ± 0.36. A similar relationship has been observed for Donors #3 and #5, who have almost the same number of IFNγ positive cells measured by flow cytometry, 23.04% and 23.34%, respectively, but the number of SFCs from Donor #5 was three-times higher compared to Donor #3: 2.9 ± 0.23 *vs.* 0.94 ± 0.12 (Figure 1 and Figure 2, Table 5). On the other hand, Donors #2 and #5 had almost the same number of SFCs measured by ELISPOT, 3.1 ± 0.36 and 2.9 ± 0.23, respectively, but Donor #2 had 30% more IFNγ-positive cells measured by ELISPOT (Table 5).

**Table 3:** Flow cytometry analysis of IFNγ-positive cells isolated from five different donors. Cells were stimulated with mitogens, as described in the Experimental Section.

Donor	Non-Stimulated Cells, %			Stimulated Cells, %		
	CD3 Negative Cells	T-cells	All Cells	CD3 Negegative Cells	T-Cells	All Cells
**1**	0.61	0.65	1.26	21.0	12.40	33.40
**2**	0.33	0.50	0.83	18.50	13.80	32.30
**3**	1.18	0.75	1.93	16.50	6.54	23.04
**4**	1.02	0.77	1.79	8.29	7.83	16.12
**5**	0.59	0.74	1.33	7.84	15.50	23.34

**Table 4:** Ranking of donors based on the capacity of their cells to produce IFNγ measured by flow cytometry.

Cells	Rankings of Donors
**Non-stimulated CD3 neg. cells**	3 > 4 > 1 > 5 > 2
**Non-stimulated T-cells**	4 > 3 > 5 > 1 > 2
**All non-stimulated cells**	3 > 4 > 5 > 1 > 2
**Stimulated CD3 neg. cells**	1 > 2 > 3 > 4 > 5
**Stimulated T-cells**	5 > 2 > 1 > 4 > 3
**All stimulated cells**	1 > 2 > 5 > 3 > 4

**Table 5:** Comparison of IFNγ-positive cells measured by flow cytometry with the number of spot-forming cells (SFCs) measured in ELISPOT. The fraction of IFNγ-secreting cells was calculated by dividing the number of IFNγ-positive cells measured by flow cytometry by the number of SFCs measured by ELISPOT and expressed as a percentage.

Donor	Stimulated Cells		Fraction of IFNγ- Secreting Cells (%)
	Flow Cytometry (“All Cells”, %)	ELISPOT (SFCs ± SD, %)	
**1**	33.4	6.52 ± 0.23	19.52
**2**	32.3	3.1 ± 0.36	9.6
**3**	23.04	0.94 ± 0.12	4.08
**4**	16.12	0.46 ± 0.02	2.85
**5**	23.34	2.9 ± 0.23	4.28

### 3.4. ELISA vs. Flow Cytometry 

It appears that, for the most part, a ranking of donors in flow cytometry did not correlate with that one in ELISA: 1 > 2 > 5 > 3 > 4 *vs.* 2 > 4 ≥ 5 > 1 > 3 respectively. Although Donor #1 had the largest number of IFNγ-positive cells (33.4%), it turned out that they were the weakest IFNγ-secreting cells (212 ± 4.38 pg/mL) measured by ELISA (Figure 2, Table 2, Table 3 and Table 5). Donors #1 and #2 had a similar number of IFNγ-positive cells (Table 5), but the amount of secreted IFNγ (Table 2) from Donor #2 was almost seven-times higher compared to Donor #1 (1,449 ± 0.0 pg/mL *vs.* 212 ± 4.38 pg/mL). Donors #4 and #5 had the same levels of secreted IFNγ, 268 ± 8.19 pg/mL and 266 ± 1.803 pg/mL, respectively (Table 2), but Donor #4 had 30% less IFNγ-positive cells than Donor #5 (Table 3).

### 3.5. Discussion

We have analyzed the expression level and secretion capacity of IFNγ from peripheral blood mononuclear cells isolated from five healthy donors in side-by-side testing using ELISPOT, ELISA and flow cytometry assays. It is well known that ELISPOT does not permit the quantification of the amount of cytokines released from the cells [8,9], although by combining it with ELISA and using complicated calculations, it is possible to get an estimate of the average amount released at the single-cell level [22]. It appears that, often, due to limited resources and time constraints, researchers choose to employ just a single technique, not realizing that the data may lead them in the wrong direction. In our study, we used two analytical approaches: (i) ranking the donors depending on IFNγ-producing and IFNγ-secreting activity of their PBMCs; and (ii) side-by-side comparison of PBMCs from individual donors that were analyzed by the ELISPOT, ELISA and flow cytometry techniques.

Our data agree with reports from other investigators: the number of positive IFNγ-producing cells measured by flow cytometry far exceeds the number of secreting cells measured by ELISPOT [23,24]. One of the explanations suggests that a significant proportion of IFNγ-positive cells may represent the cells that have bound and internalized IFNγ [24], and we hypothesize that this could be also due to a negative feedback regulation of IFNγ secretion: when the level of IFNγ secreted by a fraction of IFNγ-positive cells reaches a certain point, other IFNγ-positive cells recognize it as a “do not secrete” signal.

There was a perfect correlation of donors’ ranking between ELISPOT and flow cytometry, but not between ELISPOT and ELISA or flow cytometry and ELISA. In other words, flow cytometry predicted the overall IFNγ-secreting potency of PBMCs in ELISPOT, but not in ELISA experiments. Although it is often expected by researchers that the number of SFCs in ELISPOT should be proportional to the number of cytokine-positive cells estimated by flow cytometry and the levels of secreted IFNγ when measured by ELISA, our data do not support such an approximation. To the contrary, the results of our study suggest that a larger number of SFCs may be produced by a lower number of IFNγ-positive PBMCs and *vice versa* (Table 1 and Table 3). On the other hand, Donors #1 and #2, who had similar numbers of IFNγ-positive cells after the mitogen stimulation measured by flow cytometry showed 2–3-fold differences in the number of SFCs in ELISPOT, and Donors #2 and #5, who had the same number of SFCs measured in ELISPOT, displayed 30% differences in the number of IFNγ- positive cells measured by flow cytometry (Table 1 and Table 3). It appears that this phenomenon can be explained by individual differences between PBMCs from different donors with respect to the same stimulating mitogen: Donors #1 and #2 responded to mitogen stronger than Donors #3, #4 and #5, which have overall a larger number of non-stimulated IFNγ-positive PBMCs (compare the “all cells” columns in Table 3), and such a differential sensitivity was observed for both CD3− and T-cells (Table 3). It does not appear possible to do a true comparison between the amount of secreted IFNγ with either the number of IFNγ-positive cells or IFNγ-secreting cells, due to the fact that the IFNγ amount produced in PBMC with respect to mitogenic stimulation varies depending on which subsets of cell (NK cells, CD4+ T-cells, CD8+ T-cells) contribute to producing the IFNγ. As shown by our flow cytometry data, stimulated CD3− lymphocytes from four donors had a higher number of IFNγ-positive cells, and it is known that CD3− generally have a higher production of IFNγ than CD3+ T-cells. Unlike other investigators, who observed that the relationships between identical serum and cellular cytokines were weak, but in a positive direction [23], we found that such a direction can be negative. It will be interesting to investigate the mechanisms underlying the differential sensitivities to find out whether these mechanisms are universally applicable to all secreted cytokines regardless of mitogens and treatment conditions.

Another interesting result of our study is a lack of correlation between the number of IFNγ-secreting cells and the amount of secreted IFNγ quantified by ELISPOT and ELISA, respectively. It has been reported that ELISPOT is more sensitive than ELISA in general [24] and exceeds the sensitivity of the latter by up to 200 times [25], and it is expected that there should be a positive correlation between the number of SFCs quantified in ELISPOT and the total amount of secreted cytokine measured by ELISA. It has been reported that there is a weak, but positive correlation between serum and intracellular cytokines [23]. In our study, we observed a positive correlation in donors’ ranking between ELISPOT and flow cytometry, but not between ELISA and either ELISPOT or flow cytometry. Pronounced negative donor-to-donor correlations were found when comparing ELISA and ELISPOT: donors who secreted the same amount of IFNγ measured by ELISA showed six-fold differences in the number of SFCs measured by ELISPOT, and donors who had 5–7-times less secreted IFNγ measured by ELISA showed a two-fold increase in the number of SFCs measured by ELISPOT compared to donors who showed a more profound secretion of IFNγ measured by ELISA (Table 1 and Table 2). A negative correlation between ELISPOT and ELISA may have an important prognostic value: donors with high levels of IFNγ secreted by a small number of cells are at a higher risk of losing a significant amount of this cytokine, even if a small fraction of secreting cells is lost due to pathological or drug treatment conditions, whereas a depletion of the same percentage of “large number/low-level” cytokine secreting cells is not expected to have such a dramatic effect.

The results of our study suggest that there can be a negative donor-to-donor correlation between ELISPOT, ELISA and flow cytometry (Table 6).

**Table 6:** Normalized values from ELISPOT, flow cytometry and ELISA IFNγ measurements. The normalized data for three assays types from five donors use values obtained for flow cytometry (Table 5, “all cells” percent positive for IFNg), ELISpot (Table 5, percent SCF) and ELISA (Table 2, pg/mL). For each type of assay, the lowest value was set to 1.0, and the results with other donors are reported as multiples of that value.

Donor	Normalized Values		
	ELISPOT	Flow Cytometry(All stimulated cells)	ELISA
**1**	14.17	2.07	4.42
**2**	6.74	2.00	30.19
**3**	2.04	1.43	1.00
**4**	1.0	1.00	5.58
**5**	6.30	1.45	5.54
**Rankings of Donors**	1 > 2 > 5 > 3 > 4	1 > 2 > 5 > 3 > 4	2 > 4 ≥ 5 > 1 > 3

The higher number of cytokine-positive cells determined by flow cytometry is not necessarily indicative that there will be a high number of cytokine-secreting cells when they are analyzed either by ELISPOT or ELISA. Our ELISPOT *vs.* ELISA comparison demonstrates that the higher number of SFCs observed in ELISPOT does not necessarily indicate that these cells secrete larger amounts of cytokines compared to donors with lower SFC numbers.

## 4. Conclusions

ELISPOT, ELISA and flow cytometry are very powerful techniques for analyzing immune system cells that produce and secrete different cytokines. Although it is tempting to assume that there should be a positive correlation between the measurements obtained using different techniques, our experimental data do not support such an assumption and suggest that the correlation can be negative. It appears that using ELISPOT, ELISA and flow cytometry techniques in combination rather than as standalone applications can shed more light on the physiology of cytokine-secreting immune system cells.
